# The comparative prevalence of comorbidities across rheumatoid arthritis, psoriatic arthritis and axial spondyloarthritis

**DOI:** 10.1093/rap/rkaf121

**Published:** 2025-10-13

**Authors:** Jacob C Williams, Joshua Southworth, Kira Rogers, Sizheng Steven Zhao

**Affiliations:** St James’s University Hospital, Leeds Teaching Hospitals NHS Trust, Leeds, UK; Leeds Institute of Rheumatic and Musculoskeletal Medicine, University of Leeds, Leeds, UK; School of Medical Sciences, University of Manchester, Manchester, UK; School of Medical Sciences, University of Manchester, Manchester, UK; Centre for Musculoskeletal Research, The University of Manchester, Manchester, UK

**Keywords:** RA, PsA, axial spondyloarthritis, inflammatory arthritis, comorbidity

## Abstract

**Objectives:**

Inflammatory arthritis (IA) is associated with a high comorbidity burden, yet few studies have compared comorbidities across IA subtypes. We aimed to compare 39 comorbidities across RA, PsA and axial spondyloarthritis (axSpA).

**Methods:**

We used UK Biobank data from over 500 000 participants aged 40–69 years. Baseline data for IA and comorbidities were identified via ICD-10 codes, primary care records and/or self-report. Analysis of variance (ANOVA) and chi-squared tests were used for group comparisons, and logistic regression was used to estimate adjusted odds ratios (ORs) for comorbidities in IA versus controls.

**Results:**

Of 230 055 participants (45.4% male; mean age 56.5 years), 1969 had RA, 606 PsA and 797 axSpA. Hypertension (prevalence 9.0–11.1%) and dyspepsia (5.3–7.9%) were more prevalent in IA than in controls. Individuals with RA had significantly higher odds of atherosclerotic cardiovascular diseases, including coronary artery disease (OR 2.1, 95% confidence interval [CI] 1.66, 2.67), stroke/transient ischaemic attack (TIA) (OR 1.78, 95% CI 1.09, 2.89) and peripheral vascular disease (OR 1.90, 95% CI 1.21, 2.99). Those with axSpA had higher odds of heart failure (OR 2.37, 95% CI 1.29, 4.34), atrial fibrillation (OR 1.59, 95% CI 1.03, 2.45) and epilepsy (OR 2.33, 95% CI 1.10, 4.94).

**Conclusion:**

IA is linked to increased comorbidity prevalence, notably in cardiovascular, respiratory and gastrointestinal systems. The prevalence and odds of developing atherosclerotic cardiovascular disease were highest in those with RA. Individuals with axSpA have increased odds of developing epilepsy. These findings highlight the diverse comorbidity profiles across IA subtypes and support tailored management approaches.

Key messages

- Cardiovascular and gastroenterological comorbidities are highly prevalent across inflammatory arthritides.
- Atherosclerotic cardiovascular disease is more prevalent in RA than in other inflammatory arthritides.
- Individuals with axial spondyloarthritis have higher odds of developing epilepsy than controls.

## Introduction

Inflammatory arthritis (IA), including RA, PsA and axial spondyloarthritis (axSpA), is frequently associated with other medical conditions or comorbidities [1]. This association may be due to shared risk factors (e.g. obesity, smoking), consequences of chronic systemic inflammation (e.g. cardiovascular disease [CVD]) or the effects of its treatment (e.g. glucocorticoids leading to osteoporosis) [2, 3].

Comorbidities are highly relevant to the management of IA. A higher comorbidity burden has been linked to reduced physical function and lower health-related quality of life [4, 5]. Comorbidities can influence treatment choices, particularly in cases of chronic infection, heart failure or kidney disease [6–8]. They also affect assessments of disease activity and responses to treatment (e.g. chronic pain and osteoarthritis) [9]. Moreover, comorbidities are often the primary cause of mortality in people with IA (e.g. CVD and cancer) [10].

Most studies to date have focused on either individual comorbidities or a selection of conditions (e.g. CVD) and/or have been restricted to a single type of IA. The relative prevalence of conditions within and across the three IAs is less clear. Furthermore, many previous studies recruited IA patients from specialist and/or research settings, which may not be representative of comorbidity burden across primary and secondary care. Understanding the epidemiology of comorbidities in RA, PsA and axSpA is essential for service planning, comprehensive disease assessment and management strategies. We aimed to characterise the prevalence of 39 comorbidities in a large cohort of individuals with RA, PsA and axSpA.

## Methods

We conducted a cross-sectional study using data from the UK Biobank, a cohort of over 500 000 individuals aged 40–69 years recruited between 2006 and 2010. The UK Biobank is a research tissue bank with ethical approval by the North West Multicentre Research Ethics Committee (21/NW/0157). Participants provided informed consent for study participation, including data collection and linkage to health records. Access to the UK Biobank for this study was granted under application number 72723. For the current observational analysis, we used cross-sectional data from the baseline assessment and included only participants with linked primary care data.

Participants with RA, PsA and axSpA were identified through (1) ICD-10 codes from hospital admission records, (2) Read codes from primary care records, and/or (3) self-reported diagnoses at their baseline visit. Individuals with code for more than one IA were excluded. Individuals without IA were used as controls.

We selected comorbidities based on prior research on multimorbidity in the UK population; this study included 40 chronic diseases based on recommendations for the measurement of multiple chronic diseases by the National Health Service [11]. The list was modified by replacing psoriasis (due to its link with PsA) with eczema, removing inflammatory polyarthropathy/connective tissue disease, modifying painful conditions to chronic pain, and adding osteoporosis due to its relevance in IA. Comorbidities were defined using at least one ICD-10 code or primary care (Read) code; self-reported diagnoses were not used because they were not consistently available for all comorbidities (full code list provided in Supplementary Table S1, available at *Rheumatology* Online).

Descriptive statistics were used, with means presented for continuous variables and percentages for categorical variables. We used logistic regression to compare the odds of each comorbidity between each IA and the control group, adjusting for age, sex, body mass index (kg/m^2^; except in the obesity analysis), CRP (mg/l) and smoking status (current, previous or never). In response to peer review, we additionally adjusted for Index of Multiple Deprivation (IMD) as a marker of socioeconomic status. Analyses were performed using Stata v15.0.

## Results

Of 230 055 participants in the UK Biobank with linked primary care data, we included 1969 individuals with RA (mean age 59.6 years, SD 6.9; 29.4% male), 606 individuals with PsA (mean age 56.9 years, SD 7.3; 52.5% male), 797 individuals with axSpA (mean age 57.8 years, SD 7.5; 64.5% male) and 226 683 controls (mean age 56.5 years, SD 8.1; 45.4% male), as detailed in Table 1. Participants with RA were generally older than those with other types of IA and controls. The RA group had a higher proportion of females, while the axSpA group had a predominance of males. BMI was elevated across all IA groups (27.7–28.6 kg/m^2^), with the highest BMI observed in those with RA (28.4 kg/m^2^, SD 5.6) and PsA (28.6 kg/m^2^, SD 5.3). CRP levels were elevated in all IA groups, with particularly high levels in the RA group (6.2 mg/l, SD 9.1). Additionally, a history of former or current smoking, as opposed to never smoking, was more prevalent among individuals with IA.

**Table 1.** Prevalence of comorbidities in controls and IA (RA, PsA, axSpA)

	Controls	RA	PsA	axSpA
Total number, *n*	226 683	1969	606	797
Age in years, mean (SD)	56.5 (8.1)	59.6 (6.9)	56.9 (7.3)	57.8 (7.5)
Male sex, *n* (%)	102 882 (45.4)	579 (29.4)	318 (52.5)	514 (64.5)
BMI in kg/m^2^, mean(SD)	27.5 (4.8)	28.4 (5.6)	28.6 (5.3)	27.7 (4.8)
CRP in mg/l, mean (SD)	2.6 (4.3)	6.2 (9.1)	4.8 (7.7)	5.1 (7.6)
Non-smokers, *n* (%)	124 175 (55.1)	883 (45.1)	289 (48.1)	374 (47.2)
Ex-smokers, *n* (%)	77 525 (34.4)	841 (43.0)	256 (42.6)	320 (40.4)
Current smokers, *n* (%)	23 794 (10.6)	232 (11.9)	56 (9.3)	98 (12.4)
Cardiovascular/metabolic				
Atrial fibrillation, *n* (%)	3210 (1.4)	37 (1.9)	10 (1.7)	23 (2.9)
Coronary artery disease, *n* (%)	3848 (1.7)	81 (4.1)	19 (3.1)	24 (3.0)
Diabetes mellitus, *n* (%)	3312 (1.5)	64 (3.3)	14 (2.3)	17 (2.1)
Heart failure, *n* (%)	1032 (0.5)	22 (1.1)	4 (0.7)	11 (1.4)
Hypertension, *n* (%)	13 255 (5.8)	181 (9.2)	67 (11.1)	72 (9.0)
Obesity, *n* (%)	1960 (0.9)	40 (2.0)	12 (2.0)	6 (0.8)
Peripheral vascular disease, *n* (%)	994 (0.4)	24 (1.2)	3 (0.5)	3 (0.4)
Stroke/TIA, *n* (%)	978 (0.4)	18 (0.9)	3 (0.5)	6 (0.8)
Respiratory				
Asthma, *n* (%)	6222 (2.7)	81 (4.1)	15 (2.5)	31 (3.9)
Bronchiectasis, *n* (%)	278 (0.1)	10 (0.5)	0 (0.0)	2 (0.3)
COPD, *n* (%)	1056 (0.5)	32 (1.6)	7 (1.2)	7 (0.9)
Gastroenterological/hepatic				
Chronic liver disease, *n* (%)	610 (0.3)	11 (0.6)	5 (0.8)	2 (0.3)
Constipation, *n* (%)	975 (0.4)	20 (1.0)	6 (1.0)	10 (1.3)
Diverticulitis, *n* (%)	2428 (1.1)	34 (1.7)	12 (2.0)	15 (1.9)
Dyspepsia, *n* (%)	7737 (3.4)	155 (7.9)	32 (5.3)	48 (6.0)
Inflammatory bowel disease, *n* (%)	724 (0.3)	9 (0.5)	2 (0.3)	17 (2.1)
Irritable bowel syndrome, *n* (%)	5743 (2.5)	61 (3.1)	25 (4.1)	28 (3.5)
Viral hepatitis, *n* (%)	489 (0.2)	0 (0.0)	1 (0.2)	1 (0.1)
Genitourinary				
Chronic kidney disease, *n* (%)	540 (0.2)	8 (0.4)	1 (0.2)	8 (1.0)
Prostate disorders, *n* (%)	1228 (0.5)	12 (0.6)	7 (1.2)	9 (1.1)
Mental health/psychiatric				
Anxiety disorder, *n* (%)	1096 (0.5)	4 (0.3)	3 (0.5)	4 (0.5)
Dementia, *n* (%)	128 (0.1)	6 (0.3)	0 (0.0)	0 (0.0)
Depression, *n* (%)	3916 (1.7)	53 (2.7)	13 (2.1)	16 (2.0)
Eating disorder, *n* (%)	74 (0.03)	1 (0.1)	1 (0.2)	0 (0.0)
Schizophrenia/bipolar, *n* (%)	256 (0.1)	2 (0.1)	2 (0.3)	0 (0.0)
Substance abuse disorder, *n* (%)	226 (0.1)	3 (0.2)	0 (0.0)	1 (0.1)
Cancer, *n* (%)	16 214 (7.2)	146 (7.4)	42 (6.9)	58 (7.3)
Ophthalmological				
Blindness and low vision, *n* (%)	162 (0.1)	3 (0.2)	0 (0.0)	0 (0.0)
Glaucoma, *n* (%)	1155 (0.5)	19 (1.0)	4 (0.7)	8 (1.0)
Neurological				
Epilepsy, *n* (%)	780 (0.3)	7 (0.4)	3 (0.5)	7 (0.9)
Migraine, *n* (%)	3910 (1.7)	44 (2.2)	12 (2.0)	12 (1.5)
Multiple sclerosis, *n* (%)	275 (0.1)	4 (0.2)	0 (0.0)	0 (0.0)
Parkinson’s disease, *n* (%)	158 (0.1)	2 (0.1)	0 (0.0)	2 (0.3)
Other				
Chronic pain, *n* (%)	662 (0.3)	14 (0.7)	4 (0.7)	6 (0.8)
Deafness, *n* (%)	3127 (1.4)	42 (2.1)	14 (2.3)	18 (2.3)
Eczema, *n* (%)	3522 (1.6)	31 (1.6)	14 (2.3)	7 (0.9)
Osteoporosis, *n* (%)	1497 (0.7)	72 (3.7)	4 (0.7)	19 (2.4)
Sinusitis, *n* (%)	1063 (0.5)	15 (0.8)	6 (1.0)	5 (0.6)
Thyroid disorders, *n* (%)	4248 (1.9)	66 (3.4)	18 (3.0)	13 (1.6)

Controls defined as individuals without IA.

axSpA: axial spondyloarthritis; BMI: body mass index; COPD: chronic obstructive pulmonary disease; CRP: C-reactive protein; IA: inflammatory arthritis: n: number; PsA: psoriatic arthritis; RA: rheumatoid arthritis; SD: standard deviation; TIA = transient ischaemic attack.

The prevalence of CVD was elevated among individuals with IA, particularly those with RA. Hypertension was common across all IA types, affecting 9.0–11.1% of individuals, compared with only 5.8% of controls. Among the IA subtypes, RA demonstrated the highest prevalence of coronary artery disease (4.1%), diabetes mellitus (3.3%), peripheral vascular disease (1.2%) and stroke or transient ischaemic attack (TIA) (0.9%). In contrast, axSpA was associated with the highest prevalence of other cardiac conditions, including heart failure (1.4%) and atrial fibrillation (2.9%). Obesity was more prevalent in the RA (2.0%) and PsA (2.0%) populations, where it was approximately twice as prevalent compared with the axSpA and control groups.

Gastrointestinal symptoms, particularly dyspepsia (5.3–7.9%), were more prevalent across all IA types than controls (3.4%). Inflammatory bowel disease (IBD) was markedly more common in axSpA (2.1%) than in other IA subtypes and controls. Osteoporosis was at least three times more prevalent among individuals with RA (3.7%) and axSpA (2.4%) compared with those with PsA and controls. Depression was more common among individuals with IA, with participants with RA (2.7%) demonstrating the highest prevalence. Notably, cancer prevalence was similar between IA patients and controls, suggesting no difference in cancer risk associated with IA.

Adjusted logistic regression models demonstrated increased odds of CVD in IA. Participants with IA had increased odds of hypertension compared with controls: RA (odds ratio [OR] 1.29, 95% confidence interval [CI] 1.10, 1.53), PsA (OR 1.76, 95% CI 1.34, 2.31) and axSpA (OR 1.38, 95% CI 1.07, 1.78). The odds of atherosclerotic CVD, including coronary artery disease (OR 2.10, 95% CI 1.66, 2.67), stroke/TIA (OR 1.78, 95% CI 1.09, 2.89) and peripheral vascular disease (OR 1.90, 95% CI 1.21, 2.99), were elevated in RA. In addition, the odds for several risk factors of metabolic syndrome (MetS) and CVD were higher in RA, including diabetes mellitus (OR 1.92, 95% CI 1.47, 2.51) and obesity (OR 1.63, 95% CI 1.17, 2.28). Individuals with PsA tended towards atherosclerotic heart disease with increased odds of coronary artery disease (OR 1.66, 95% CI 1.03, 2.68), whereas those with axSpA tended towards other cardiac diseases, with higher odds of heart failure (OR 2.37, 95% CI 1.29, 4.34) and atrial fibrillation (OR 1.59, 95% CI 1.03, 2.45). The odds of chronic kidney disease were uniquely increased in individuals with axSpA (OR 3.29, 95% CI 1.62, 6.70).

The odds of asthma (OR 1.31, 95% CI 1.04, 1.67), bronchiectasis (OR 2.82, 95% CI 1.47, 5.40) and COPD (OR 2.04, 95% CI 1.40, 2.98) were increased in individuals with RA. Among gastrointestinal comorbidities, dyspepsia showed higher odds across IA groups: RA (OR 2.06, 95% CI 1.73, 2.45), PsA (OR 1.47, 95% CI 1.02, 2.11) and axSpA (OR 1.57, 95% CI 1.16, 2.13). Irritable bowel syndrome (IBS) had higher odds in PsA (OR 1.72, 95% CI 1.15, 2.57) and axSpA (OR 1.57, 95% CI 1.07, 2.29) compared with controls, and the odds of IBD were increased 6-fold in axSpA (OR 6.13, 95% CI 3.75, 10.01).

The odds of osteoporosis were elevated in RA (OR 3.98, 95% CI 3.08, 5.14) and axSpA (OR 4.51, 95% CI 2.79, 7.28) compared with controls. Individuals with RA also had higher odds of depression (OR 1.41, 95% CI 1.06, 1.87) and dementia (OR 4.18, 95% CI 1.81, 9.70).

The odds of ophthalmological and neurological comorbidities were low in the cohort, with only the odds of glaucoma (OR 1.69, 95% CI 1.07, 2.68) in RA and epilepsy (OR 2.33, 95% CI 1.10, 4.94) in axSpA being elevated. There were no increased odds of cancer in any IA when compared with controls.

**Figure 1.**
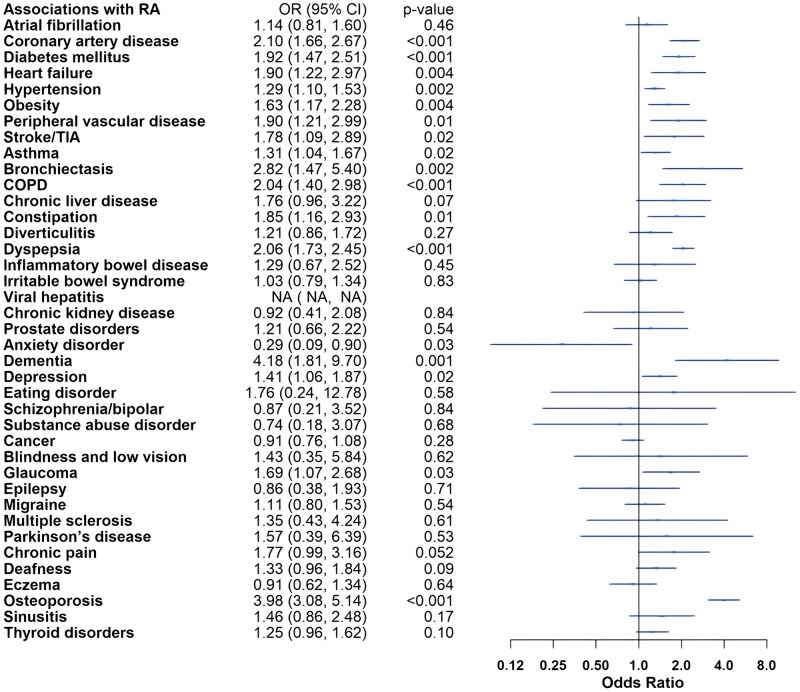
Adjusted logistic regression models for rheumatoid arthritis compared with non-IA controls. COPD: chronic obstructive pulmonary disease; TIA: transient ischaemic attack

**Figure 2.**
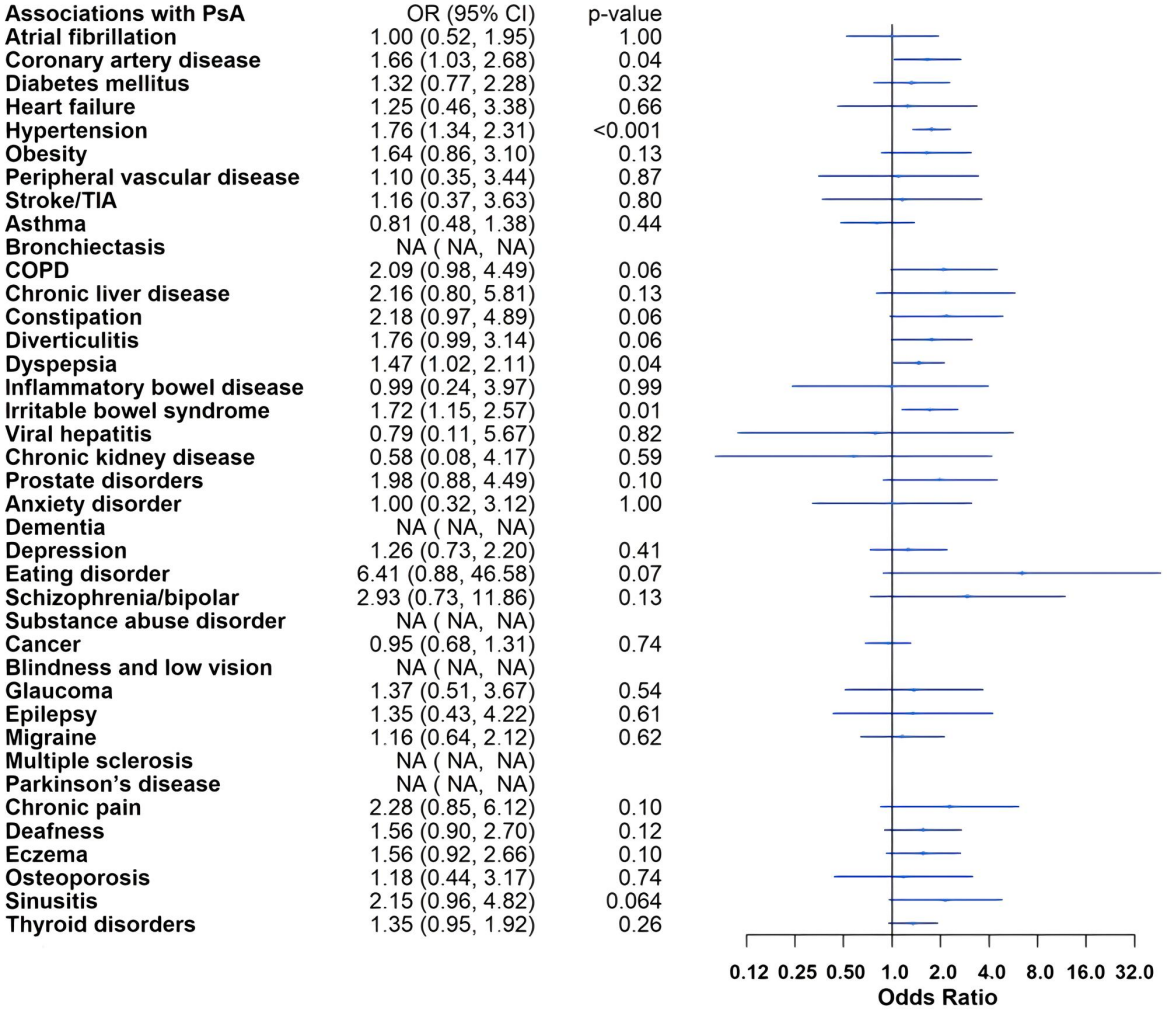
Adjusted logistic regression models for psoriatic arthritis compared with non-IA controls. COPD: chronic obstructive pulmonary disease; TIA: transient ischaemic attack

**Figure 3.**
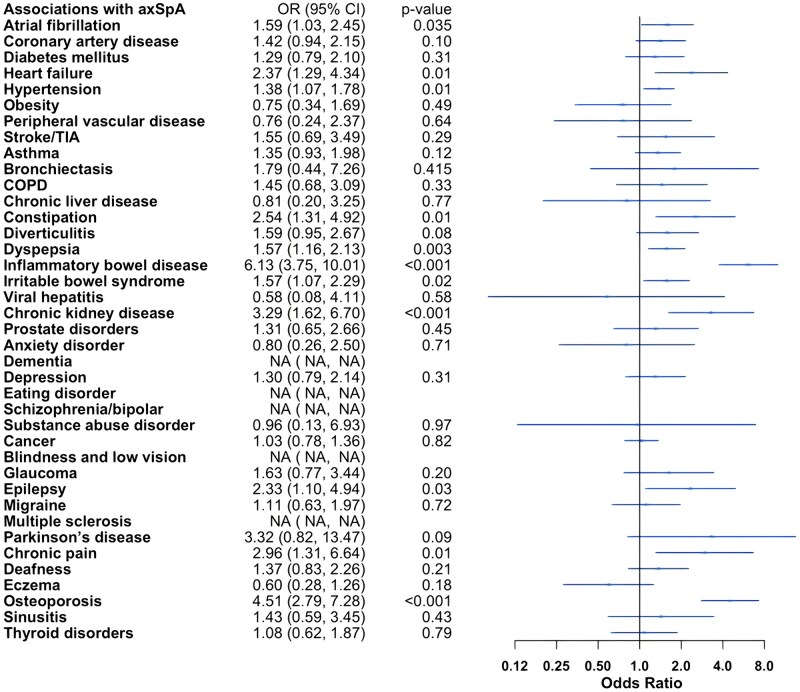
Adjusted logistic regression models for axial spondyloarthritis compared with non-IA controls. axSpA: axial spondyloarthritis; COPD: chronic obstructive pulmonary disease; TIA: transient ischaemic attack

Full adjusted model results are presented in Figs 1–3. Sensitivity analysis additionally adjusting for IMD did not meaningfully change results (data not shown).

## Discussion

In this large cross-sectional study, IA was associated with a high prevalence of comorbidities compared with controls, particularly cardiovascular, respiratory and gastroenterological conditions. The prevalence and odds of developing atherosclerotic CVD were highest in those with RA, while axSpA patients were more prone to other cardiac disorders, such as atrial fibrillation and heart failure. Hypertension and dyspepsia were prevalent across all types of IA, but the distribution of other comorbidities varied by IA type. Finally, we found increased odds of epilepsy in axSpA compared with controls, a finding that has been rarely reported previously and warrants replication. Our findings reiterate the high burden of comorbidities in individuals with IA and highlight novel differences in the specific comorbidities affecting each subtype.

Our findings for RA are consistent with existing literature, demonstrating a propensity for cardiovascular, metabolic and mental health disorders, along with a higher prevalence of respiratory disorders, such as COPD and bronchiectasis, and primary open-angle glaucoma [4, 12–17]. Although our results suggested lower odds of anxiety in RA, this likely reflects a spurious finding given the low number of cases due to under-reporting of anxiety diagnoses, a trend previously noted in individuals with RA [18]. Anxiety and depression tend to co-occur, yet anxiety was 10-fold less common than depression in the current RA data. We also showed increased risk of dementia, and although the overall event number was low across groups, this is in keeping with previous large-scale studies showing increased risk of Alzheimer’s disease in RA (OR 2.06; 95% CI 2.02, 2.10) [19]. Similarly, cardiac and metabolic disorders—including hypertension, ischaemic heart disease, heart failure, atrial fibrillation, stroke and hyperlipidaemia—along with depression, are among the most frequently reported comorbidities in axSpA [3, 20]. Our analysis corroborates these findings, showing an increased prevalence of hypertension, atrial fibrillation and heart failure in axSpA. Although an increased cardiovascular risk, including atherosclerosis, in axSpA is well recognized, more recent studies have demonstrated a comparatively increased risk of atherosclerotic CVD in RA compared with axSpA [3, 21–24]. Our findings suggest the magnitude of the risk of atherosclerotic CVD may differ between RA, PsA and axSpA. As axSpA does not characteristically present with high levels of systemic inflammation, this may explain the difference in risk [3]. Osteoporosis, inflammatory bowel disease, irritable bowel syndrome and peptic ulcer disease have also been reported at higher prevalences in axSpA, a trend reflected in our analysis [3, 20, 25–27]. Interestingly, we have reported increased odds of epilepsy in axSpA. Watad *et al.* [28] also reported an association between epilepsy and axSpA in their Israeli cohort, with an OR of 2.33 (95% CI 1.75, 3.09). As neurological disorders have typically been underrepresented in previous comorbidity research in axSpA, our findings may represent an unrecognized comorbidity in axSpA [3]. Equally, because an association with epilepsy has rarely been reported in the literature, it should be interpreted with caution pending future studies. The mechanism underlying this association is unclear, and event counts were low overall; the focus of future research should be to confirm this association and delineate the reasons responsible for it. The association may be explained by shared risk factors (e.g. smoking) or consequences of axSpA symptoms (e.g. sleep disturbance lowering seizure thresholds), although these are highly speculative.

For PsA, the literature indicates an increased prevalence of cardiometabolic comorbidities, including obesity, hypertension, MetS and CVD [2, 20, 29, 30]. A meta-analysis by Loganathan *et al.* [29] found a higher prevalence of MetS in PsA compared with RA. Notably, although not statistically significant, the frequency of obesity was consistent in both PsA and RA groups (2.0%), which was double that of controls (0.9%). The mean BMI (kg/m^2^) was highest in the PsA group (PsA 28.6 vs RA 28.4 vs control 27.5).

The reported prevalence of comorbidities in our study is substantially lower than in prior literature, likely reflecting the generally middle-aged, healthier and more affluent cohort utilized in the UK Biobank [2, 3, 12, 29, 31, 32].

The mechanisms underlying multimorbidity in IA are thought to be multifactorial. Factors contributing to CVD and atherosclerosis in IA include chronic systemic inflammation, adverse effects of disease-modifying antirheumatic drugs (DMARDs), such as hypertension from leflunomide and dyslipidaemia from Janus kinase inhibitors, and reduced physical function and activity, compounded by other cardiovascular risk factors (e.g. hypertension, obesity, smoking) [2, 3, 33–36]. Dyspepsia is frequently secondary to anti-inflammatory therapies, including NSAIDs, corticosteroids and several DMARDs [37].

Our findings emphasize the importance of identifying and managing comorbidities in IA. The management of cardiometabolic comorbidities, especially hypertension, is relevant across RA, PsA and axSpA. Rheumatologists should collaborate with primary care physicians to optimize the management of comorbidities, with particular emphasis on cardiovascular and gastroenterological diseases. This may involve dietary interventions, lifestyle modifications, medical management or surgical interventions as appropriate [1, 5, 38]. Clinicians should consider both arrhythmia and epilepsy in patients with axSpA who present with transient loss of consciousness. Although our study suggests that there may be differences in cardiovascular manifestations between RA, PsA and axSpA, more evidence is required before considering IA-specific recommendations for cardiovascular screening.

Our study has several strengths. It is one of the first to compare a large number of important chronic comorbidities between RA, PsA and axSpA. We utilized a large sample size from an established national cohort study with over 230 000 individuals recruited from the general population, rather than many prior studies that recruited IA participants from specialist and/or research centres, and thus may better represent comorbidity burden across primary and secondary care.

Our study also has several limitations. Participants in the UK Biobank are generally recognized as being healthier than the UK population [32]. Despite this, we detected a high degree of comorbidity within individuals with IA, suggesting this may be an even greater issue in rheumatology clinical practice. Additionally, as self-reported and previously coded data were used, there is the possibility of misclassification. However, we minimized misclassification across the three IAs by excluding individuals with codes for more than one IA. Prior studies of PsA in the UK Biobank showed that this case definition had high genetic correlation with CASPAR-criteria PsA cases, suggesting misclassification is likely limited [39]. Inclusion of non-inflammatory cases should generally bias estimates towards the null. Even if classification criteria data were available, it would preferentially select patients with more established disease who are likely to have a higher comorbidity burden. Similarly, since data on disease activity and disease duration were unavailable, we were unable to adjust for or explore further associations with these variables. Additionally, we lacked access to accurate medication data to adjust our analysis. We were not able to estimate the incidence of these comorbidities, which would provide important context for prevalence estimates and should be a focus of future research.

In summary, our findings indicate a high burden of comorbidity in individuals with IA, particularly among those with RA. Future large cohort studies should aim to investigate the prevalence of comorbidities in axSpA, PsA and RA, with a focus on identifying key differences among different forms of IA, particularly regarding patterns of cardiovascular risk and the relationship between axSpA and epilepsy.

## Supplementary Material

rkaf121_Supplementary_Data

## Data Availability

UK Biobank data are available to all bona fide researchers for use in health-related research that is in the public interest. The application procedure is described at www.ukbiobank.ac.uk
