# Supplementary material for: The comparative prevalence of comorbidities across rheumatoid arthritis, psoriatic arthritis and axial spondyloarthritis
Source: Rheumatol Adv Pract. 2025 Oct 13;9(4):rkaf121. doi: 10.1093/rap/rkaf121 (PMC12571505; doi:10.1093/rap/rkaf121)
Supplement: rkaf121_Supplementary_Data [file rkaf121_supplementary_data.docx]

**Supplementary Table S1: Definitions of conditions using ICD-10 and primary care read codes**

| Condition | Read Codes | ICD Codes |
| --- | --- | --- |
| Anxiety disorder | Eu4 | F40-F48 |
| Axial spondyloarthritis | N100, N11F | M081, M45 |
| Asthma | H33 | J45 |
| Atrial fibrillation | G573 | I48 |
| Blindness and low vision | F49 | H54 |
| Bronchiectasis | H34 | J47 |
| Cancer | B | C, D |
| Chronic liver disease | J61-J63 | K7 |
| Chronic pain | R00z2, R00zC | R52 (excluding R52.0), M79.7 |
| Chronic kidney disease | K05 | N18 |
| Constipation | J520 | K59.0 |
| Chronic obstructive pulmonary disease (COPD) | H3 (excluding H30, H33, H34, H35) | J44 |
| Coronary artery disease | G3 | I20-I25 |
| Deafness | F59 | H90-H91 |
| Dementia | E00 (excluding E00y, E00z), Eu00-Eu02 | F00-F03 |
| Depression | Eu32-Eu33 | F32-F33 |
| Diabetes mellitus | C10 | E10-E14 |
| Diverticulitis | J51 | K57 |
| Dyspepsia | J11-J17, J1y.., J1z.. | K20, K30-K31 |
| Eating disorder | Eu50 | F50 |
| Eczema | M11 | L20 |
| Epilepsy | F25 | G40-G41 |
| Glaucoma | F45 | H40, H42 |
| Heart failure | G58 | I50 |
| Hypertension | G20, G24-G26, G28 | I |
| Irritable bowel syndrome | J51, J521 | K58 |
| Inflammatory bowel disease | J40-J41 | K50-K51, K52.3 |
| Migraine | F26 | G43 |
| Multiple sclerosis | F20 | G35 |
| Obesity | C38 | E66 |
| Osteoporosis | N330-N331 | M80-M82 |
| Parkinson’s disease | F12 | G20 |
| Peripheral vascular disease | G73 | I73 (excluding I73.0) |
| Prostate disorders | K20 | N40 |
| Psoriatic arthritis | M160 | M07.0-M07.3, L40.5 |
| Rheumatoid arthritis | N040-N042 | M05-M06 (excluding M06.1) |
| Schizophrenia/manic psychosis | Eu2, Eu30-Eu31 | F2, F30-F31 |
| Sinusitis | H13 | J32 |
| Stroke/TIA | G65-G66 | G45-G46 |
| Substance misuse disorder | Eu11-Eu19 | F11-F19 |
| Thyroid disorders | C02, C04 | E03, E05 |
| Viral hepatitis | A70 | B15-B19 |
